# Stretchable Capacitive Pressure Sensing Sleeve Deployable onto Catheter Balloons towards Continuous Intra-Abdominal Pressure Monitoring

**DOI:** 10.3390/bios11050156

**Published:** 2021-05-14

**Authors:** Kirthika Senthil Kumar, Zongyuan Xu, Manivannan Sivaperuman Kalairaj, Godwin Ponraj, Hui Huang, Chi-Fai Ng, Qing Hui Wu, Hongliang Ren

**Affiliations:** 1 Department of Biomedical Engineering, National University of Singapore, Singapore 117575, Singapore; s.kirthika@u.nus.edu (K.S.K.); zongyuan.xu@u.nus.edu (Z.X.); biesikm@nus.edu.sg (M.S.K.); godwin.joseph@u.nus.edu (G.P.); 2 Singapore Institute of Manufacturing Technology, A*STAR, Singapore 138634, Singapore; hhuang@simtech.a-star.edu.sg; 3 S.H. Ho Urology Centre, Department of Surgery, Prince of Wales Hospital, The Chinese University of Hong Kong, Hong Kong 999077, China; ngcf@surgery.cuhk.edu.hk; 4 Department of Urology, National University Hospital, Singapore 119074, Singapore; qing_hui_wu@nuhs.edu.sg; 5 Department of Electronic Engineering, Faculty of Engineering, Chinese University of Hong Kong, Hong Kong 999077, China; 6 Shun Hing Institute of Advanced Engineering, The Chinese University of Hong Kong (CUHK), Hong Kong 999077, China

**Keywords:** biomedical monitoring, IAP monitoring, pressure sensor, sensing sleeve, soft sensors

## Abstract

Intra-abdominal pressure (IAP) is closely correlated with intra-abdominal hypertension (IAH) and abdominal compartment syndrome (ACS) diagnoses, indicating the need for continuous monitoring. Early intervention for IAH and ACS has been proven to reduce the rate of morbidity. However, the current IAP monitoring method is a tedious process with a long calibration time for a single time point measurement. Thus, there is the need for an efficient and continuous way of measuring IAP. Herein, a stretchable capacitive pressure sensor with controlled microstructures embedded into a cylindrical elastomeric mold, fabricated as a pressure sensing sleeve, is presented. The sensing sleeve can be readily deployed onto intrabody catheter balloons for pressure measurement at the site. The thin and highly conformable nature of the pressure sensing sleeve captures the pressure change without hindering the functionality of the foley catheter balloon.

## 1. Introduction

**Figure 1:**
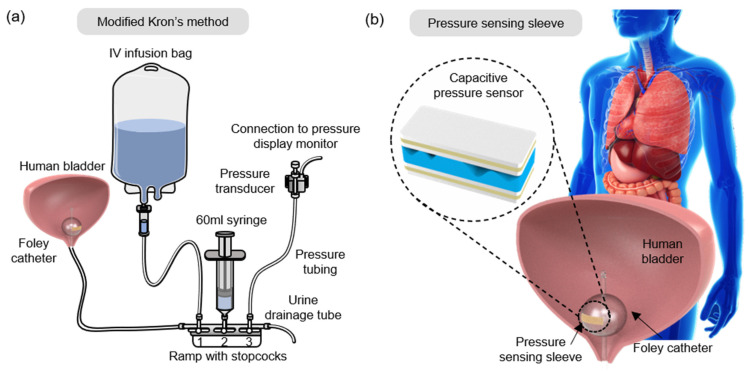
Measurement set-up for intra-abdominal pressure (IAP) monitoring. (**a**) Schematic overview of modified Kron’s method for IAP measurements. (**b**) Proposed slip-on pressure sensing sleeve for continuous IAP measurements.

Intra-abdominal pressure (IAP) is the pressure within the abdominal cavity enclosed by the abdominal wall and the viscera. Normal IAP is defined to be below 12 mmHg. Sustained elevation of IAP > 12 mmHg (1.6 kPa) is termed as intra-abdominal hypertension (IAH), and elevation >20 mmHg (2.7 kPa) is defined as abdominal compartment syndrome (ACS). These IAP elevations can lead to conditions altering organ perfusion and organ dysfunctions [1,2], or even organ failure in extreme cases [3]. Regular monitoring of IAP aids in the early diagnosis and management of IAH and ACS, which, in turn, has been proven to reduce the rate of morbidity and mortality [1,2,4]. As recommended by the World Society of the Abdominal Compartment Syndrome (WSACS), IAP measurements are to be made every four hours for critically ill patients. The gold-standard measurement of IAP is via the transvesical route, whereby the measurement system (pressure transducer) is connected to a standard urinary catheter and a urinary drainage tube, as shown in Figure 1a [5,6,7]. The steps involved in the gold-standard measurement (modified Kron’s method) [8] are detailed in Table 1.

However, this pressure measurement process is labor-intensive, with a long preparation time for a single-point measurement [9]. In addition, the placement of a sensor far from the pressure source and the risk of air bubbles in the measurement tube pose limitations. The inability to capture early signs of an elevated IAP and to provide immediate treatment increases the mortality rates up to 55%. However, timely recognition can reduce the mortality rate by almost 5%. Therefore, a continuous IAP monitoring is superior to the current indirect intermittent measurement methods by providing timely recognition of the significant changes in IAP. Furthermore, with the ever-increasing medical cases and workload on healthcare workers, automation of crucial measurement parameters will ease labor-intensive procedures and better utilize the workforce and resources. Thus, there is a need for an efficient and continuous way of measuring IAP [10]. Ingestible capsules have been designed for continuous IAP measurements. However, this requires sophisticated fabrication facilities and leaves the possibility of capsule retention in the digestive tract of patients [11]. There have been advancements in sensor development, such as integrated circuit-based pressure sensors through intra-vaginal IAP measurement [12]. Other advancements include microfluidic device-based ultrasonic imaging, which is more invasive and requires the sensor to be implanted [13], as well as the use of tensiometry to measure abdominal wall tension [14]. 

Catheters instrumented with directly mounted pressure sensors have been on the rise over the years, leading to promising outcomes [15,16]. Several ways of fabricating pressure sensors [17] have emerged from studies for various applications [18,19,20,21,22,23]. Typical sensing techniques involve the measurement of resistance [24], voltage [25], or capacitance [26]. These transduction principles are also adopted to make flexible and stretchable pressure sensors greatly relevant to biomedical applications [27]. In particular, capacitance-based sensors typically have a good sensitivity even at low-pressure settings (<40 mmHg) [28]. A supercapacitor-based sensor was deployed on the Foley catheter tube to measure the distributed urethral pressure [29]. However, no effort was made to deploy a stretchable pressure sensor directly on the catheter balloon for direct IAP measurements. In recent developments, microstructures such as pillars, pyramids, and hemispheres have been used to bring a change in the dielectric volume between the electrodes, boosting its sensitivity [30]. Alternatively, pores can be introduced into the dielectric material by introducing randomly distributed pores using a foaming agent [31] or phase separation [32,33]. Controlled distribution of the pores can be done through the sacrificial solvent method [34,35] or electrospinning to form nanofibers [36]. The microstructure or porous structure increases the mechanical compressibility of the sensors and therefore increases the response rate of the pressure sensors by mitigating the viscoelasticity and hysteresis effects. However, these methods are often associated with a high cost or complex fabrication process [37].

The main contributions of the work are as follows:

(1) A potential method of fabrication altering the sensitivity for capacitance-based pressure sensors.
(2) Incorporation of a pressure sensor into a stretchable pressure sensing sleeve.
(3) Introduction of a pressure sensing sleeve on a Foley catheter for continuous monitoring of the intra-abdominal pressure.

This paper presents a pressure sensing sleeve fabricated for IAP monitoring (Figure 1b). Its sensitivity under low-pressure ranges (<2.7 kPa) is clinically relevant for IAP monitoring and the early diagnosis of IAH and ACS. The stretchable nature of the sensing sleeve accommodates catheter balloon inflation. Furthermore, the thickness of the sensor and the sleeve is designed to be as thin as possible, in accordance with the diameter of the urethral opening. Thus, it behaves as an add-on feature, readily deployable to any commercial Foley catheters of various sizes, without additional catheter changes. The sleeve is designed to monitor IAP continuously, with a significant decrease in the tediousness of the process. The materials chosen for fabrication have good compatibility and workability for IAP monitoring. Microstructures were introduced into the dielectric material of the sensor by pattern transfer from abrasive papers of various grit (particle/grain) sizes. This characterization serves as the basis of optimization of the sensor on the grit sizes and thickness of the dielectric material. The proposed system has good sensitivity and aims to incorporate additional functionality of pressure sensing into already existing catheters. This could potentially reduce the labor intensiveness of the current practice.

## 2. Materials and Methods

### 2.1. Pressure Sensor Fabrication

**Figure 2:**
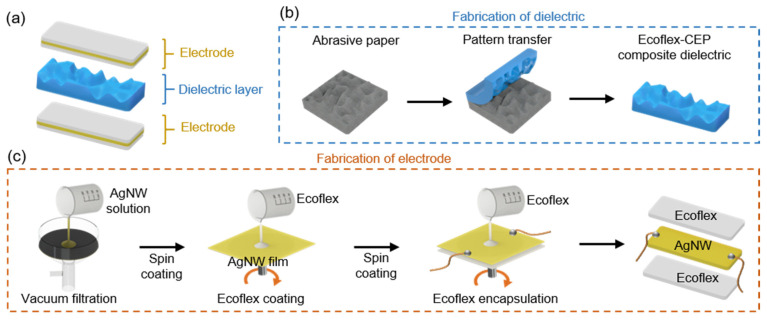
Schematic representation IAP pressure sensor: (**a**) components and sandwich layer structure of the pressure sensor, and the fabrication process of the (**b**) dielectric and (**c**) electrode.

The stretchable pressure sensor comprises two electrode layers and a dielectric layer (Figure 2a). The dielectric layer was obtained by a composite mixture of Ecoflex (Smooth-On, Inc., Macungie, PA, USA) and cyanoethyl pullulan (CEP) (Shin-Etsu Chemical Co., Ltd., Japan) suspension in a 4:1 ratio. The CEP suspension consists of 4.5 g of CEP in 16 g of propylene carbonate (PC) (Sigma-Aldrich, Inc., St. Louis, MO, USA). The Ecoflex–CEP composite was spin-coated onto an abrasive paper by filling it into the abrasive paper’s voids. Upon curing, the abrasive paper was peeled off, leaving the irregularly-patterned microstructures transferred onto the Ecoflex–CEP dielectric layer (Figure 2b). Electrodes were fabricated through a layer-by-layer scalable assembly process (Figure 2c). The silver nanowire (AgNW) suspension was dripped evenly onto a filter paper placed under vacuum filtration in order to obtain a uniform thin AgNW film layer. The obtained filter paper with an AgNW film had a sheet resistance of ~5 Ω/sq. It was then spin-coated with a two-part Ecoflex solution at 1000 rpm for 30 s. Upon curing of the Ecoflex, the filter paper substrate was peeled off, and AgNWs were dry transferred onto the Ecoflex substrate. The sheet resistance of the Ecoflex infiltrated AgNW film was ~9.46 Ω/sq. This change in sheet resistance had a negligible effect on its use as an electrode. Wire connections were made on the exposed side of the AgNW layer and were then spin-coated with Ecoflex again to obtain an encapsulated electrode. This resulted in an electrode with a thickness of 600 µm.

### 2.2. Sensing Sleeve Fabrication

**Figure 3:**
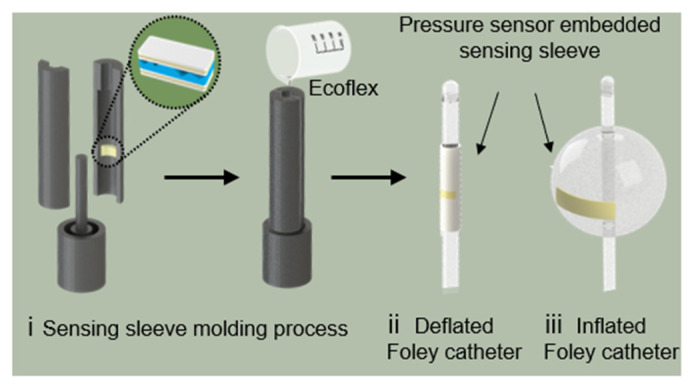
Pressure sensing sleeve on a Foley catheter: Schematic representation of the fabrication of the pressure sensing sleeve.

The measurement of IAP through the Bard 1236-14 Foley catheter balloon (C.R. Bard, Inc., New Providence, NJ, USA) requires the assembled pressure sensor to be incorporated into a sensing sleeve, allowing for the easy instrumentation of the catheter with a sensing capability. Hence, the assembled pressure sensor was sealed in a diaphragm to minimize the stretching and preserve air voids. The diaphragm was designed to be slightly larger than the fabricated pressure sensor to provide space for the air pocket and to allow for the adaptation of the pressure sensors when encountering high pressure. The diaphragm comprised two layers of cured Ecoflex (thickness = 200 µm). These Ecoflex layers were pre-stretched, and the pressure sensor was placed between them. A small tube connected to a vacuum pump was inserted to remove air from the diaphragm before sealing it with uncured Ecoflex along with the corners. The pressure sensor encapsulated by the diaphragm was then transferred onto a custom-designed 3D printed PLA mold, as conceptualized in Figure 3. A balloon sleeve incorporating the sensor was obtained through injection molding with Ecoflex. The pressure-sensing sleeve was designed to accommodate the inflation of the catheter balloon (Figure 3).

## 3. Results and Discussion

### 3.1. Dielectric Optimization

**Figure 4:**
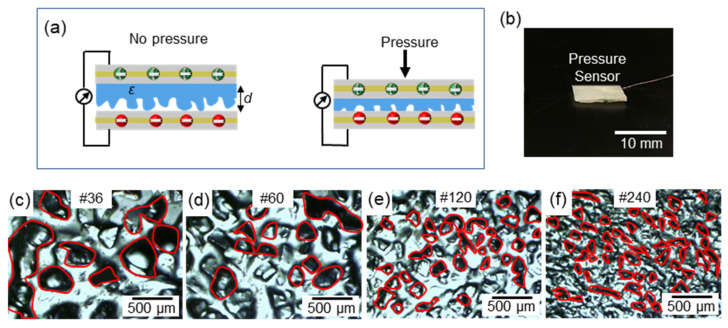
Dielectric material optimization with geometric variations. (**a**) Sensing principle of the sensor with microstructures upon applied pressure with varying distance (*d*). (**b**) IAP capacitive pressure sensor. Optical microscopic images of random microstructures of dielectric where some of the air voids are marked in red for (**c**) grit #36, (**d**) grit #60, (**e**) grit #120, and (**f**) grit #240.

**Figure 5:**
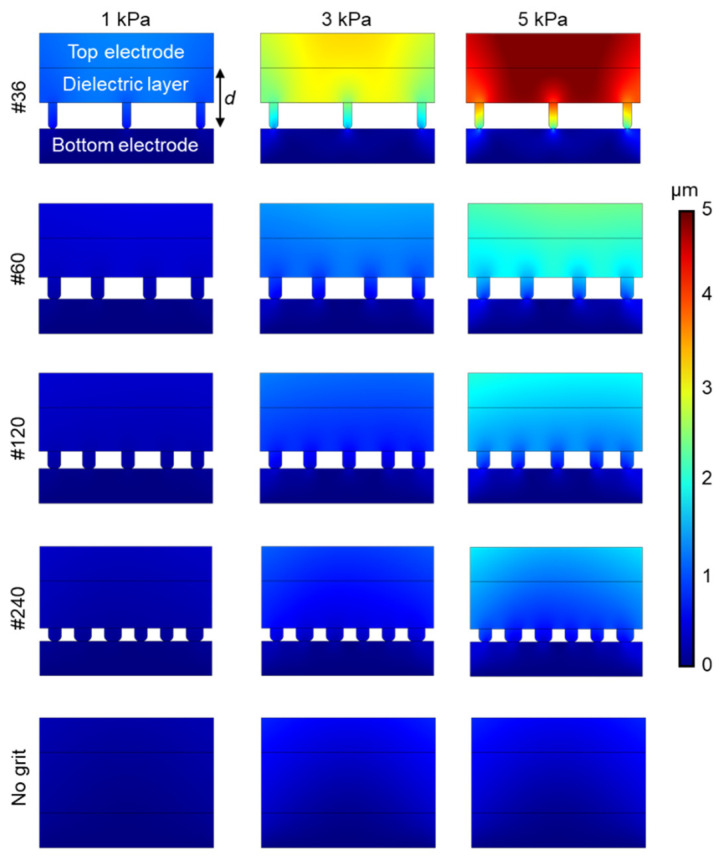
Finite element analysis performed on different grit sizes showing the change in distance between the electrodes for applied pressure.

**Figure 6:**
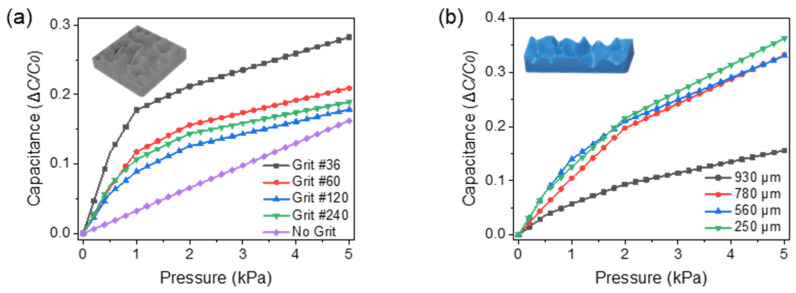
Dielectric material optimization with geometric variations. (**a**) Sensor response to the effect of dielectric microstructure modification. (**b**) Sensor response to varied dielectric thicknesses.

The sensor works on the principle of transducing pressure into capacitance, as illustrated in Figure 4a. Upon pressure, the capacitance (*C*) increases (i) due to the reduction in distance (*d*) between the two electrodes and (ii) the change in the dielectric constant (*ε*) between the electrodes. Figure 4b shows the fabricated capacitive pressure sensor as per the procedure mentioned in Figure 2. Modifications on the geometry of the dielectric layer have been proven to improve sensor performance [38,39]. To evaluate this, the Ecoflex-CEP composite was spin-coated on abrasive paper of different grit sizes. The optical microscopy images are shown in Figure 4c–f, which was taken with an IX51 microscope (Olympus, Tokyo, Japan) equipped with a DP72 digital imaging system (Olympus) and Olympus LUC Plan FLN 20×/0.45 and 10×/0.30 objective lens (Olympus, Japan). COMSOL Multiphysics^®^ 5.3 Simulation Software (COMSOL Inc., Burlington, MA, USA) was used to estimate the dependence of the different grit sizes on the change in distance (*d*) between the two electrodes after 1, 3, and 5 kPa of applied pressure. Each grit size had variations in the size and distance between its microstructures. From Figure 5, it can be observed that for the same amount of pressure, the change in distance between the electrodes was higher for grit #36 compared with the other grit sizes and the sensor without any grit. This is because of the compressibility and low modulus (~125 kPa) of the Ecoflex elastomer in the dielectric layer. As mentioned earlier, a larger change in distance between the electrode layers induces a larger capacitance change. The capacitance variation (Δ*C/C*_0_) to the pressures of dielectrics fabricated with various grit sizes is shown in Figure 6a. In the dielectric with no grit (bulk), the applied pressure was converted to internal stress. By adding microstructures, air voids were created, which increased the sensors’ mechanical compressibility, leading to greater changes in *d* for smaller pressures. Furthermore, a change in the dielectric media in the space is expected because of the displaced air volume (lower dielectric constant). From the experimental results, larger microstructures (Grit #36) were observed to have a better sensitivity to pressure compared with smaller (Grit #240) or no microstructures.

Furthermore, the effect of the thickness of the dielectric media was studied (Figure 6b). The Ecoflex–CEP composite was spin-coated on abrasive paper of grit #36 at different rpm to obtain dielectric of various thicknesses. The thickness of the dielectric film was measured using a IL series CMOS Multi-Function Analogue Laser Sensor (Keyence, Japan), which was calibrated to the base distance between the laser and the dielectric elastomer to be measured. Then, the dielectric elastomer was moved across through the ridges of the random microstructures. The average value was taken as the thickness of the dielectric. From the results (Figure 6b), it can be observed that thicknesses <780 µm have a higher sensitivity (capacitance change with respect to pressure change) in the lower pressure range (<4 kPa), corresponding to the proposed application. Henceforth, dielectric media with microstructures transferred from the abrasive paper of grit #36 with a thickness <780 µm were used for the sensor.

The quantitative analysis of the relationship between the applied pressure and change in capacitance can be derived. Owing to the microstructural changes in the dielectric, the sensor exhibits two distinct linear ranges—low-pressure (*P* < 1 kPa) and higher-pressure (1 kPa < *P* < 5 kPa). Their linear relationship in the low-pressure range can be attributed to (1), with an R^2^ correlation of 0.98. Similarly, the linear relationship in the higher-pressure ranges conforms to (2), with an R^2^ correlation of 0.99.

Δ*C/C_0_* = 0.1782*P* + 0.0127 (1)

Δ*C/C_0_* = 0.0253*P* + 0.1582 (2)

Despite the non-linear characteristics in the IAP working range, the results show that the applied pressure was still directly correlated with the capacitance change, closely following a second-order polynomial of Equation (3) with an R^2^ correlation of 0.97.

Δ*C/C_0_* = −0.0354*P*^2^ + 0.1695*P* + 0.0255 (3)

### 3.2. In-Vitro Characterizations

**Figure 7:**
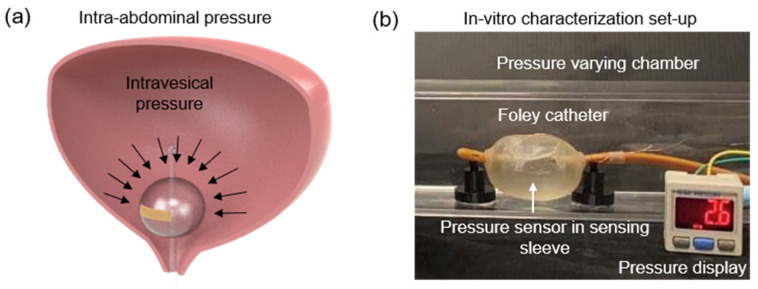
In-vitro sensor characterizations. (**a**) Pressure sensing concept. Correlation between IAP and IVP intravesical pressure (IVP). (**b**) Photograph of the pressure varying chamber set-up.

**Figure 8:**
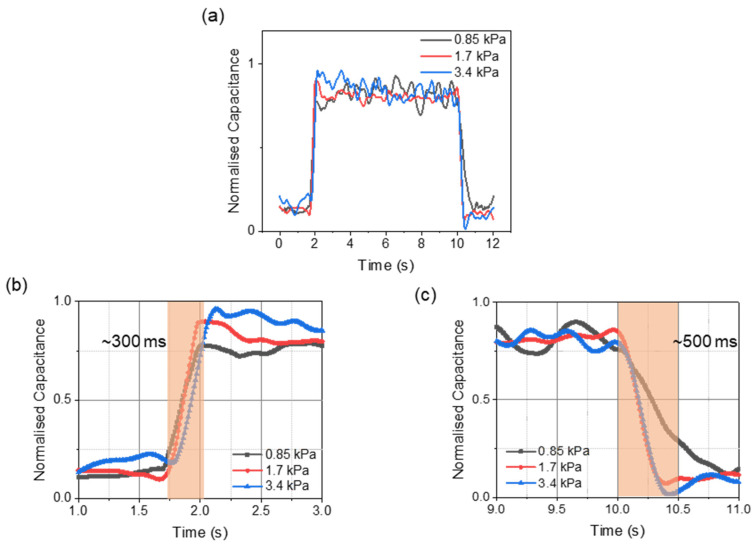
In-vitro sensor characterizations. (**a**) Response time characterization under clinically relevant pressure settings. (**b**) Rise-time of the pressure sensor. (**c**) Fall-time of the pressure sensor.

**Figure 9:**
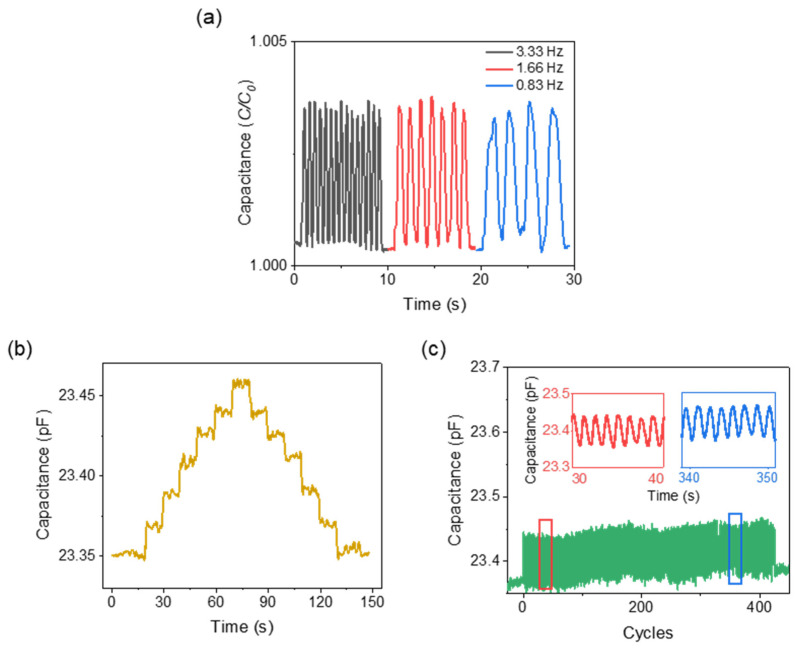
In-vitro sensor characterizations. (**a**) The sensor response to pressure change is applied in various frequencies. (**b**) Drift response of the sensor with incremental and decremental small pressure. (**c**) Cyclic loading performance evaluation of the sensor showing a negligible drift up to 400 cycles. Insets show the obtained pressure cycles in the initial and final stages.

The pressure measured in the bladder and abdomen was labeled as intravesical pressure (IVP) and intra-abdominal pressure (IAP). A close correlation exists between the measured IVP and IAP [40]. Hence, the pressure-sensing concept is such that a change in IAP will induce a change in intravesical pressure (IVP) in the human bladder, altering the measured capacitance of the proposed sensor in the Foley catheter balloon (Figure 7a). For the in-vitro characterization, experiments were performed in a varying pressure chamber that mimics the human bladder, and the readings were validated with a commercial pressure sensor (Figure 7b). The fabricated pressure-sensing sleeve was deployed onto a 14 Fr Foley catheter balloon and was inflated with 35 mL of saline solution. Figure 8a shows the response time of the pressure sensor after the application of pressure. The rise time (~300 ms) and drop time (~500 ms) remained consistent at varied pressures (Figure 8b,c). Fast provocative maneuvers like coughing or sneezing caused sudden changes in abdominal pressure. This required the sensor to capture the pressure changes instantaneously in real-time. The proposed sensors demonstrated robustness over the various air frequencies introduced into the chamber, with no deterioration at the varied frequencies (Figure 9a). To validate the effective and reliable operation of the sensor, it is necessary to ensure the sensor readings are maintained at different pressure conditions. For this purpose, the drift characteristics of the sensor over time were performed by raising the pressure by a small factor and allowing it to reside. For every change in pressure caused by a small volume change (Δ*V* = 0.0083%) in the chamber, the corresponding sensor response is demonstrated in Figure 9b. The stability of the sensor to capture the periodic change in pressure is investigated through cyclic actuation. The sensor displays its durability and reliability during cyclic pressure loading for >400 cycles (Figure 9c).

## 4. Conclusions

In this work, we presented a stretchable pressure sensing sleeve, readily deployable on Foley catheter balloons for continuous IAP monitoring. We focused on optimizing the capacitive sensor to a clinically relevant pressure range (0 kPa–4 kPa) and on integrating the sensor into a sleeve. The workability of the sensing sleeve was demonstrated with a bench-top pressure varying model. Our sensing sleeve will potentially reduce the manpower and time required to monitor patients’ IAP continuously. For post-operative patients with a prophylactic open abdomen strategy, our device could be an alternative to reduce the need for follow-up surgery.

Future work will focus on enabling the sensing sleeve to be wireless. A radio-frequency identification-based (RFID) device can extend the sensors’ signals through an antenna. This can transmit the capacitance change wirelessly to a receiver antenna outside the body [28]. Alternatively, the device could be designed to have a small battery-powered integrated circuit (IC) to collect and transfer data via wireless communication protocols such as WiFi, bluetooth, or near-field communication (NFC) [41]. However, both of these options have design complications in terms of size, form factor, etc., that need to be explored. In addition, we seek to explore other application areas such as resuscitative endovascular balloon occlusion of the aorta (REBOA). REBOA is a temporary occlusion of the aorta to prevent excessive blood loss during hemorrhage. However, there are several hazards in this procedure, namely: over-inflation of the balloon may damage the aortic wall, while slow deflation may result in profound hypotension [42,43]. Hence, our sensing sleeve can be used to perform precise monitoring of the blood pressure to ensure proper occlusion. It will also enable the use of partial REBOA to prevent a lack of blood flow to the extremities. Furthermore, the sensor’s advantages can be well utilized for intra-renal pelvic pressure monitoring during endoscopic surgery [44,45].

## Figures and Tables

**Table 1:** Steps involved in performing IAP measurements.

Steps	Current Practice
1	A ramp with three stopcocks is connected to the drainage tubing of the Foley catheter.
2	An IV infusion bag, 60 mL syringe, and a pressure transducer are connected to the ramp.
3	The bladder and the system are flushed with normal saline.
4	The pressure transducer is fixed at the top of the patient’s symphysis pubis bone or thigh.
5	Zero-point calibration of the pressure transducer is done upon stabilization.
6	Urine drainage tubing is clamped.
7	Bladder filled with <25 mL saline solution.
8	The IVP value is recorded as shown on the monitor.

## Data Availability

The data is available based on reasonable requests to the corresponding author.
